# Neurobiological and behavioural outcomes of biofeedback-based training in autism: a randomized controlled trial

**DOI:** 10.1093/braincomms/fcab112

**Published:** 2021-05-27

**Authors:** Olivia Surgent, Douglas C Dean, Andrew L Alexander, Olga I Dadalko, Jose Guerrero-Gonzalez, Desiree Taylor, Emily Skaletski, Brittany G Travers

**Affiliations:** 1 Waisman Center, University of Wisconsin-Madison, Madison, WI 53705, USA; 2 Neuroscience Training Program, University of Wisconsin-Madison, Madison, WI 53705, USA; 3 Pediatrics, University of Wisconsin-Madison, Madison, WI 53792, USA; 4 Medical Physics, University of Wisconsin-Madison, Madison, WI 53705, USA; 5 Psychiatry, University of Wisconsin-Madison, Madison, WI 53719, USA; 6 Occupational Therapy Program in Kinesiology, University of Wisconsin-Madison, Madison, WI 53706, USA

**Keywords:** neuroplasticity, autism, postural stability

## Abstract

The human brain has demonstrated the power to structurally change as a result of movement-based interventions. However, it is unclear whether these structural brain changes differ in autistic individuals compared to non-autistic individuals. The purpose of the present study was to pilot a randomized controlled trial to investigate brain, balance, autism symptom severity and daily living skill changes that result from a biofeedback-based balance intervention in autistic adolescents (13–17 years old). Thirty-four autistic participants and 28 age-matched non-autistic participants underwent diagnostic testing and pre-training assessment (neuroimaging, cognitive, autism symptom severity and motor assessments) and were then randomly assigned to 6 weeks of a balance-training intervention or a sedentary-control condition. After the 6 weeks, neuroimaging, symptom severity and motor assessments were repeated. Results found that both the autistic and non-autistic participants demonstrated similar and significant increases in balance times with training. Furthermore, individuals in the balance-training condition showed significantly greater improvements in postural sway and reductions in autism symptom severity compared to individuals in the control condition. Daily living scores did not change with training, nor did we observe hypothesized changes to the microstructural properties of the corticospinal tract. However, follow-up voxel-based analyses found a wide range of balance-related structures that showed changes across the brain. Many of these brain changes were specific to the autistic participants compared to the non-autistic participants, suggesting distinct structural neuroplasticity in response to balance training in autistic participants. Altogether, these findings suggest that biofeedback-based balance training may target postural stability challenges, reduce core autism symptoms and influence neurobiological change. Future research is encouraged to examine the superior cerebellar peduncle in response to balance training and symptom severity changes in autistic individuals, as the current study produced overlapping findings in this brain region.

## Teaser

Surgent et al. found that 6 weeks of biofeedback-based videogame balance training led to improved balance, decreased autism symptom severity and significant white matter microstructural changes across the brain in autistic and non-autistic adolescents. These findings were above-and-beyond that observed in adolescents randomly assigned to a sedentary videogame training.

## Graphical Abstract

**Graphical Abstract:**
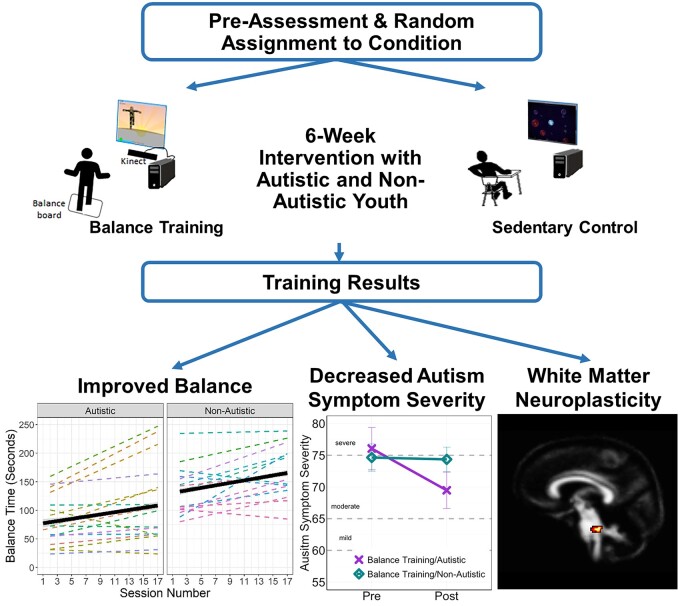

## Introduction

The past two decades of neuroscience research have revealed the power of the human brain to structurally change as a result of motor interventions.^1–7^ However, little is known regarding how the brain changes as a result of motor training in autistic individuals. (Identity-first language will be used for this paper^8^ in consideration of the preference of those in this diagnostic population^9^.) Motor impairments are highly prevalent in autism spectrum disorders (ASD),^10–12^ and motor difficulties have been linked to both more severe autism symptoms^13–15^ and poorer execution of daily living skills (DLSs).^16^^,^^17^ Owing to these motor challenges, there is a strong need to develop and rigorously test motor interventions for autistic individuals. Therefore, the purpose of the present study was to use a randomized controlled trial (RCT) design to investigate brain, balance, symptom severity and DLS changes that result from a biofeedback-based balance intervention in autistic and non-autistic adolescents.

Our team developed a 6-week, biofeedback-based, videogame training that targets balance in autistic children and adolescents.^18^ We targeted balance because postural stability challenges and atypical development of postural control are commonly reported in ASD.^19^^,^^20^ Specifically, autistic individuals demonstrated an earlier plateau in postural control development during adolescence that persisted into adulthood, as compared to non-autistic peers.^20^ Therefore, the age range of the current study (13–17 years old) was selected with the goal of preventing or ameliorating this plateau. In a previous quasi-experimental study of this training in autistic youth (ages 7–17 years), we observed significant improvements in balance, measured by both in-game progress and by postural sway measures outside of the game.^18^ In addition, participants reported that this training was beneficial and enjoyable, suggesting that autistic individuals may be likely to utilize this training in the future. While these quasi-experimental results are promising, there is a strong need for a more rigorous study design to further test the efficacy of this training and potential neuroplasticity effects.

To address this need, the present RCT had three objectives. The first objective was to rigorously test whether biofeedback-based balance training improved balance in individuals randomly assigned to the training condition (autistic *n* = 17; non-autistic *n* = 16) compared to individuals randomly assigned to a sedentary-control condition that was matched on key features (autistic *n* = 17; non-autistic *n* = 14). Based on previous research,^18^ we hypothesized that those who received the balance intervention would demonstrate greater balance improvements than those in the sedentary-control condition. Follow-up analyses examined if there were greater balance gains in autistic participants compared to non-autistic participants over the course of the 6-week training.

The second objective of the study was to identify which brain structures would demonstrate changes as a result of the balance training. Based on previous evidence indicating changes in brain structure and function following motor activity,^7^^,^^21^^,^^22^ we hypothesized that neuroplasticity would occur over the course of balance training. Specifically, we hypothesized that microstructure of the corticospinal tract (CST), an early-developing white matter (WM) motor pathway that runs from the motor and somatosensory cortices to the spinal cord, would be a likely candidate for training-based changes. Therefore, we identified the CST’s fractional anisotropy (FA), a measure of WM microstructure, as our primary outcome measure. In non-autistic individuals, fitness during adolescence has been associated with improved WM microstructure of the CST,^23^ and physiological measures of corticospinal function were enhanced after balance training.^5^^,^^24^ Furthermore, this tract has particular relevance to an autistic population, as the inferior portion of the CST has been previously linked to motor challenges and symptom severity in ASD.^25^

The third and final aim was to examine whether balance training improved core autism symptoms and DLSs compared to the sedentary-control group. Although there is evidence of relationships among motor challenges, core autism symptom severity and DLSs,^14–17^ it is unclear if motor challenges causally impact autism symptoms and poorer DLSs, or whether these associations appear correlated due to a yet-unknown third variable. This study offers a unique window into the potential cause–effect nature of these relationships by changing motor skills and measuring if those changes impact symptom severity or DLSs. Based on the notion that motor challenges may be a barrier to social communication and DLSs, we hypothesized that balance training would lead to changes in symptom severity and increased DLSs.

## Materials and methods

### Design, randomization and blinding

**Figure 1:**
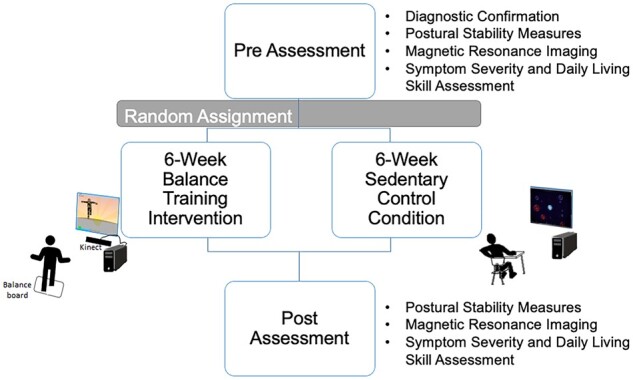
**Overview of the study procedures.** Procedure included pre-training assessment, random assignment to the balance-training or sedentary-control conditions, and post-training assessment.

This pilot study was a parallel form RCT pre-registered at clinicialtrials.gov (#NCT02358317) and designed to test superior efficacy of the biofeedback-based balance video game compared to the sedentary control condition. Pre-specified primary outcome variables included: balance times, centre of pressure (COP) measurements from Wii Balance Board and FA of the bilateral CST. Pre-specified secondary outcome variables included: adaptive DLSs and autism symptom severity. Autistic and non-autistic participants underwent a pre-training evaluation (neuroimaging, cognitive, symptom severity and motor assessments) and then underwent equal random assignment (by the corresponding author) within each diagnostic group, (1:1 for two groups) to either the biofeedback-based balance videogame training intervention or the sedentary videogame control condition (Fig. 1). At the training’s end, neuroimaging, symptom severity and motor assessments were repeated. Given the nature of the videogames, it was not feasible to blind participants or research assistants to group status. However, participants and their families were informed that the purpose of this study was to test brain and behavioural changes from videogame play, and that they would be randomly assigned to one of two types of video games. An *a priori* power calculation determined that each group (balance group/autistic, balance group/non-autistic, control group/autistic and control group/non-autistic) should have at least 13 participants, suggesting that the current sample size was sufficient (see Supplementary methods).

### Participants

The study was approved by the University of Wisconsin-Madison Institutional Review Board (IRB #2014–1499). All parents provided written informed consent, and all adolescents provided informed assent. All participants were recruited through fliers in the community, the Waisman Center registry database and word of mouth. Participants were required to be 13.0–17.9 years old. Autistic participants were required to have a prior clinical diagnosis of ASD. The prior diagnosis was supported by meeting criteria for ASD on Modules 3 or 4 of the Autism Diagnostic Observation Schedule, 2nd edition^26^ or was supported by meeting criteria for ASD on both the Social Communication Questionnaire^27^ and the Social Responsiveness Scale, 2nd edition (SRS-2)^28^ at the pre-training visit. Participants were excluded if they had a diagnosis of tuberous sclerosis, fragile X syndrome, a history of severe head injury, or hypoxia-ischemia, as the brain mechanisms of these co-occurring conditions may be unique. All participants were required to have an IQ > 60 in order to follow verbal directions, which was assessed using the Wechsler Abbreviated Scale of Intelligence, 2nd edition.^29^ At the study start, participants could not be engaged in more than 2 h/week of balance training activities (i.e. yoga, tai chi, Wii/Kinect balance games) and were asked to not start any new exercise or treatment programs during the training period. Participants were excluded if they were unable to complete the magnetic resonance imaging (MRI) scan, due to either orthodontia that might affect the quality of the scan or a contraindication to MRI (i.e. metal in the body, pacemaker, etc.). Non-autistic participants were additionally required to not have a previous diagnosis of ASD or a first-degree relative with an ASD diagnosis, as motor challenges have been reported in first-degree relatives of autistic individuals.^30^^,^^31^

### Interventions

#### Biofeedback-based balance videogame training

The procedures for the biofeedback-based balance videogame training are detailed in previous work^18^ and expanded upon in Supplementary methods. Briefly, the game was designed to increase static balance by training the participant to hold six different poses (three two-footed poses and three one-footed poses) from tai chi and yoga while providing visual feedback on the screen of how accurately the pose is being held. Custom, in-house software provided feedback via an integrated Microsoft Kinect camera and a Nintendo Wii balance board to monitor balance and posture.

#### Sedentary videogame control

Participants randomly assigned to the sedentary-control condition completed 6 weeks of playing sedentary video games selected to approximate the graphic simplicity and tempo of the intervention games. The sedentary games included *Osmos* (https://www.osmos-game.com/ Accessed 06 March 2021) and *Flow* (https://thatgamecompany.com/flow/ Accessed 06 March 2021) and required the participant to sit in a chair and use a mouse pad positioned on a desk. To mimic the maximum time that a person could hold each posture in the balance-training condition, participants in the sedentary-control condition were required to alternate playing *Flow* and *Osmos* in 240-s increments. The sedentary-control condition was carefully matched to the videogame-intervention condition on key attributes (i.e. pace of graphics, lab environment and research assistants; See Supplementary methods for more detail).

### Primary outcome measures

#### Balance time and sedentary videogame performance

As in previous work,^18^ progress during balance training was operationally defined as increased balance times in one-footed and two-footed balance poses during each session of the training.

#### Pre-post postural stability measures

As in previous work,^18^ postural sway area [i.e. area of ellipse (mm^2^) formed by COP that contains 95% of the data^32^] was assessed at baseline and at the last training session under three different conditions: (i) eyes-open standing; (ii) eyes-closed standing; and (iii) visual-feedback standing (i.e. seeing one’s COP on the screen) (full details in Supplementary methods).

#### Diffusion weighted image (DWI) acquisition and preprocessing

Whole-brain imaging data were acquired on a 3 T Discovery MR750 scanner (Waukesha, WI) at the University of Wisconsin-Madison’s Waisman Center with a 32-channel phased array head coil (Nova Medical, Wilmington, MA). DWI scans were acquired using a multi-shell spin-echo echo-planar imaging pulse sequence (6 directions at b = 0 s/mm^2^, 9 directions at b = 350 s/mm^2^, 18 directions at 800 s/mm^2^ and 36 directions at b = 2000 s/mm^2^; repetition time/echo time = 8575/76.6 ms; 2 × 2 × 2 mm^3^ isotropic voxel resolution; 128 × 128 acquisition matrix). A B_0_ field map was collected with matching geometry for use for unwarping EPI distortions due to magnetic field inhomogeneity.^33^

DWI data were preprocessed using a combination of tools from MRtrix3^34^ and FSL (version 6.0^35^; full description in the Supplementary methods). Diffusion Imaging in Python (DIPY)^36^ was used to fit diffusion tensors at each voxel within a whole-brain mask with weighted least squares to generate FA maps. All images underwent visual inspection for quality control. Additionally, participant head motion during DWI acquisition was quantified using the root mean square movement summary from *eddy.*^37^ Delineation of the bilateral CST on the DWI images was done using TractSeg, a tool that utilizes a pre-trained convolutional neural network to create bundle-specific tractograms^38^ from which we calculated mean FA across the entire CST as well as across 20 equally distanced segments along the streamlines^39^; https://github.com/MIC-DKFZ/TractSeg/. See Supplementary methods for more detail.

### Secondary outcome measures

#### Autism symptom severity

The SRS-2^28^ is a 65-item parent/caregiver questionnaire assessing the presence of autism symptoms over the past 6 months. Each item on the scale asks about an aspect of observed reciprocal social behaviour and is rated on a scale from ‘0’ (never true) to ‘3’ (almost always true). Higher scores on the SRS-2 indicate greater severity of autism symptoms.

#### Daily living skills

Two measures of DLS were used in the present study: the Vineland Adaptive Behavior Scale, 2nd edition (VABS-II)^40^ and the Waisman Activities of Daily Living Scale (W-ADL).^41^ The VABS-II is a parent-report measure of adaptive behaviours from birth to adulthood. For the purpose of the present study, only the DLS domain was used. The W-ADL is a 17-item parent/caregiver report on DLSs. For each item, the caregiver is asked to rate their child’s current level of independence in performing activities of daily living (0 = does not do at all, 1 = does with help or 2= independent or does on own). Higher scores on the VABS-2 DLS and W-ADL represent more advanced DLSs.

### Statistical analysis

#### Postural stability outcomes

Postural stability was measured by (i) in-game balance time at each training session and (ii) pre-post differences in postural sway area on the balance board outside the game. As in previous work,^18^ in-game training progress was measured by fitting linear mixed-effects models for each individual’s performance of one-footed and two-footed poses, accounting for the repeated measurements over the training sessions (see Supplementary methods). One-sample *t*-tests were used to show that the training progress was significantly greater than 0. A secondary goal was to determine whether performance during training was similar in the autistic and non-autistic groups. Therefore, independent-samples *t*-tests were performed comparing the estimated starting points and training gains between autistic and non-autistic individuals.

To test the differential effects of training on the pre-post differences in postural stability, we performed a 2 (pre/post) × 2 (balance/control) × 3 (eyes-opened/eyes-closed/visual feedback) mixed ANOVA with COP ellipse area as the dependent variable. The COP ellipse area had a positively skewed distribution, which we corrected for in analyses by using a natural log transformation. To determine whether the trainings were equally beneficial in both the autistic and non-autistic groups, we performed a follow-up 2 (pre vs. post) × 2 (balance vs. control) × 2 (autistic vs. non-autistic) × 3 (eyes-opened vs. eyes-closed vs. visual feedback) mixed ANOVA.

#### Symptom severity and DLSs outcomes

To test the differential effects of trainings on the secondary outcome measures, we performed 2 (pre/post) × 2 (balance/control) mixed ANOVA’s. Follow-up analyses were performed that added a factor to examine diagnostic group, leading to a 2 (pre/post) × 2 (balance/control) × 2 (autistic/non-autistic) mixed ANOVA.

#### Imaging outcome analyses

To test whether the CST changed according to the intervention, we fit linear mixed-effects models rather than performing ANOVA as they allowed us to better account for pre- *and* post-training changes in head motion. We analyzed the bilateral CST as a whole, using average CST FA as a function of time (pre/post), balance training group (training/control), and their interaction, while controlling for age, sex and random effects for intercepts due to repeated measures. After the null results of the primary analysis, follow-up analyses divided the tract into 20 distinct segments (from superior to inferior) and also delineated the left from the right tract.^39^ Follow-up analyses of the CST used fdr to control for multiple comparisons. See Supplementary methods for more detail.

#### Ancillary analyses

To help clarify the findings of our pre-planned analyses (#NCT02358317), we performed follow-up exploratory voxel-based analyses. For these analyses, we derived neurite orientation dispersion and density imaging (NODDI) metrics^42^ from our multi-shell DWI data. While the DTI metric of FA does not make assumptions about underlying biophysical tissue properties and can be influenced by a variety of neurobiological factors,^43^^,^^44^ NODDI metrics allow for more specific interpretation of neural structure^43^^,^^45^ via biophysical multi-compartmental modelling to quantify diffusion characteristics. NODDI metrics have been shown to be reproducible in longitudinal samples^46^ and have been validated against histological measures of neurite (e.g. axon and dendrite) structure,^47^^,^^48^ demonstrating NODDI’s superior ability to capture nuances of neurite architecture compared to classical DTI models. The NODDI-based measure of intracellular volume fraction (ICVF) is sensitive to the neurite density, while the orientation dispersion index (ODI) assesses the angular variation of the neurite orientation in a given voxel. With the preprocessed multi-shell DWI, we fit the NODDI model with the Watson distribution using Dmipy Toolbox to construct ODI and ICVF parameter maps.^49^

To determine where in the brain ODI and ICVF were associated with balance intervention-related changes we implemented the tissue-specific, smoothing compensated (T-SPOON) method for voxel-based analysis (VBA)^50^ with linear mixed-effects models. We chose the T-SPOON methodology because this technique compensates for some of the common pitfalls of VBA, thus reducing potential confounds and enhancing overall interpretability of VBA results.^50^ Linear mixed-effects models allowed us to implement more sophisticated, repeated-measures models that account for biological sex, head motion at each scan and age.

T-SPOON corrected ODI and ICVF maps were generated in accordance with previous work,^50^ except we implemented the *Atropos* tool from ANTs^51^ for WM segmentation in native space, as it has been suggested to produce more accurate segmentation than the originally used FSL *fast* algorithm.^52^ Voxel-wise statistical parametric mapping was done in R (version 4.0.2). A linear mixed-effects model was constructed in each voxel where the NODDI parameter (ICVF or ODI) was regressed on training group (intervention/control), time (pre/post), diagnosis (autistic/non-autistic), each of their two-way interactions and their three-way interaction, while accounting for sex, age and head motion. Multiple comparisons correction occurred through voxel-wise fdr-correction (*P* < 0.05) and a cluster threshold (*k*) of at least five contiguous voxels.

Because planned analyses found that balance training decreased autism symptom severity, additional follow-up voxel-based analyses explored clusters of ICVF and ODI changes as a function of changes in autism symptom severity (SRS-2). These analyses examined pre-post change in ODI or ICVF masks as a function of pre-post changes in SRS-2 raw scores, while accounting for sex, age and pre-post averaged head motion. However, these analyses were limited to the autistic participants, omitting a person who was an extreme outlier (*n *=* *16). Because of this reduction in sample size and the exploratory nature of these analyses, we used an uncorrected *P*-value of 0.005 (alongside a cluster threshold of five contiguous voxels).

### Data availability

The data that support the findings of this study are available from the corresponding author, upon reasonable request.

## Results

### Participant flow and baseline data

Recruitment and enrolment for this study occurred between May 2015 and June 2019. Participant flow can be seen in Supplementary Fig. 1. Enrolled participants included 34 community-ascertained autistic adolescents (ages 13–17 years; three females) and 30 community-ascertained non-autistic adolescents (ages 13–17 years; six females). Within the autistic group, ASD diagnoses for 30 participants were supported via the Autism Diagnostic Observation Scale, 2nd edition and ASD diagnoses for an additional four participants were supported by meeting criteria on both Social Communication Questionnaire and SRS-2. Pre-training, groups were well-matched on age (*P* = 0.48). However, we found lower IQ in the autistic sedentary-control group compared to the non-autistic sedentary-control group. Body mass index (BMI) also significantly differed (*P* = 0.02), with follow-up analyses suggesting higher BMI in the autistic balance group compared to both non-autistic groups. See Table 1 for more detailed baseline participant demographics. No adverse events occurred in this study.

**Table 1:** Demographic information after random assignment to balance-training or sedentary-control group

	Autistic balance training (*n* = 17)	Autistic control (*n* = 17)	Non-autistic balance training (*n* = 15)	Non-autistic control (*n* = 13)	ANOVA *F*	ANOVA *P*-value	*Post hoc* results
Sex, %Male	94%	88%	73%	85%	—	—	—
Age, mean (SD)	15.6(1.27)	15.44(1.35)	15.08(1.42)	14.87(1.59)	0.83	0.48	—
Age, range	13.15–17.85	13.01–17.51	13.24–17.69	13.12–17.83	—	—	—
FSIQ, mean (SD)	106.06(18.09)	101.82(17.36)	110.73(9.64)	117.46(14.67)	2.74	0.05	Non-autistic control > autistic control
FSIQ, range	73–136	67–131	92–130	96–136	—	—	—
VIQ, mean (SD)	104.76(18.45)	97.47(17.90)	109.33(12.52)	109.46(14.50)	1.9	0.14	—
VIQ, range	71–130	69–143	85–130	80–134	—	—	—
PIQ, mean (SD)	106.59(20.33)	106.18(18.37)	109.4(9.36)	123.92(20.50)	3.1	0.03	Non-autistic control > autistic control and autistic balance
PIQ, range	81–147	70–130	94–132	92–160	—	—	—
BMI, mean (SD)	27.11(7.13)	24.66(5.54)	22.03(4.67)	21.38(2.54)	3.65	0.02	Autistic balance > non-autistic
BMI, range	16.82–40.35	15.13–39.45	16.53–30.13	17.39–25.06	—	—	—

#### Primary outcome: Balance times

**Figure 2:**
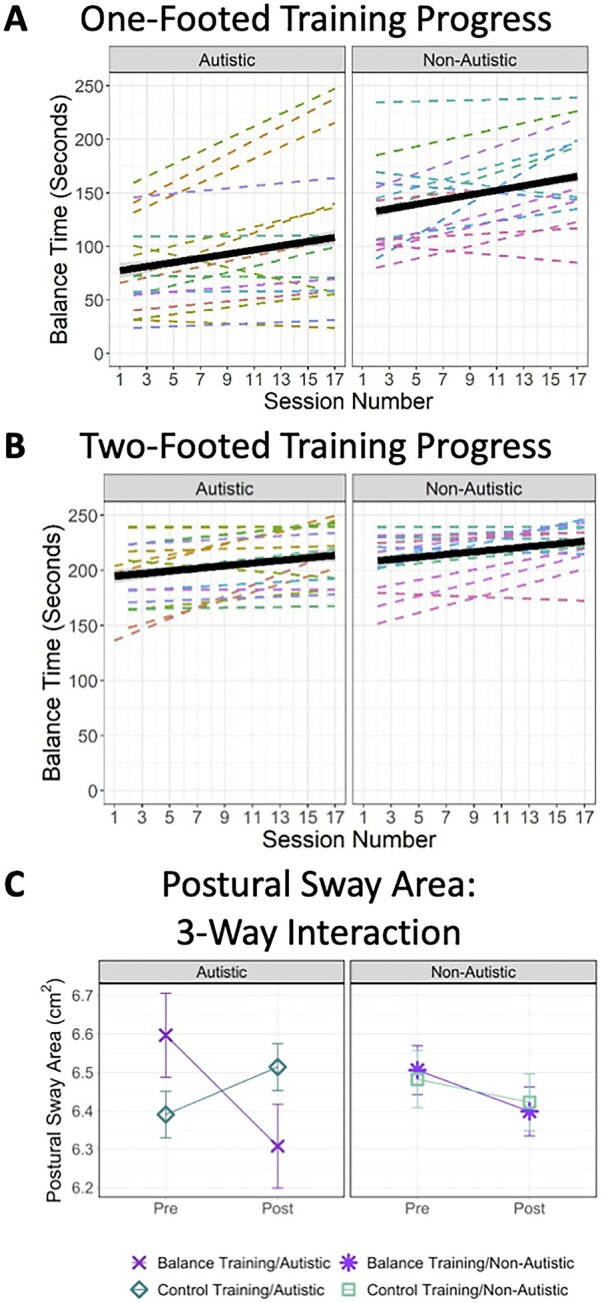
**Findings from the primary outcome measure of postural stability.** (**A**) one-footed balance times during training, (**B**) two-footed balance times during training, and (**C**) natural log transformed postural sway area measures both pre- and post-training in autistic participants compared to non-autistic participants. A and B show fitted linear smoothed lines for balance improvements over the course of the training sessions at both the level of the individual participants (dashed lines) and at the level of the group (black solid lines). Overall, the participants demonstrated significant training progress in both one-footed and two-footed poses (*P’*s < 0.001), and there were no significant group differences in the slope of training progress for either one-footed or two-footed poses (*P’*s ≥0.85). C depicts the postural sway area means (natural log transformed) ± one standard error. The hypothesized balance-training-specific decreases in postural sway area were observed.

For one-footed poses (Fig. 2A), participants started the training by being able to hold poses for an average of 101.93 s (*SD* = 49.52). However, there was a significant difference in the starting balance times by diagnostic group, such that the autistic group started at an average of 77.60 s (*SD* = 40.96), and the non-autistic group started at an average of 129.50 s (*SD* = 44.50), *t*(30) = 3.43, *P* = 0.002. Looking at training progress, participants were able to increase their ability to hold one-footed balance postures by 2.02 seconds each session (*SD* = 2.45). A one-sample *t*-test, showed that this gain was significant, *t*(31) = 4.68, *P* < .001. The autistic group (*M *=* *1.95 s, *SD* = 2.54) and non-autistic group (*M *=* *2.11 s, *SD* = 2.42) did not differ in the rate of balance improvements over the course of training, *t*(30) = 0.19, *P* = 0.85.

For two-footed poses (Fig. 2B), participants started out almost at the training ceiling (240 s) with an average start time of 199.59 s (*SD* = 31.07). The two-footed starting balance times did not differ between the diagnostic groups, *t*(30) = 1.17, *P* = .25. Even though starting near ceiling, participants were able to increase their ability to hold two-footed balance postures by 1.20 s each session (*SD* = 1.46). A one-sample *t*-test, showed that this gain was significant, *t*(31) = 4.66, *P* < 0.001, and an independent-sample *t*-test showed that this gain did not differ between the autistic participants (*M *=* *1.24 s, *SD* = 1.55) and non-autistic participants (*M *=* *1.15 s, *SD* = 1.39), *t*(30) = −0.17, *P* = 0.87.

#### Primary outcome: Postural sway area

As can be seen in Table 2, there was not a significant three-way interaction between training group, pre-post, and type of measurement of postural sway area, *F*(2,58) = 1.14, *P* = 0.32, $\eta_{p}^{2}=0.019$, confirming that pre-post changes were similar across all three types of postural sway in our analysis. There was a significant two-way interaction between training group and pre-post measure, *F*(1,59) = 4.42, *P* = 0.04, $\eta_{p}^{2}=0.070$. The three-way interaction from the follow-up mixed ANOVA that included diagnostic group further clarified that the pre-post improvements in postural sway were similar in the autistic and non-autistic groups, *F*(1,57) = 2.45, *P* = 0.12, $\eta_{p}^{2}=0.041$ (Fig. 2C). Significant terms from all analyses indicated medium to large effect sizes.^53^

**Table 2:** ANOVA results for primary outcome of postural sway area, not considering diagnostic status (left) and considering diagnostic status (right)

Postural sway: 2 × 2 × 3 ANOVA								Postural sway: 2 × 2 × 3 × 2 ANOVA						
Predictor	*df*	Sum of squares	Mean square	*F*	*P*	$\eta_{p}^{2}$		Predictor	*df*	Sum of squares	Mean square	*F*	*P*	$\eta_{p}^{2}$
Pre-post	(1,59)	0.60	0.60	1.94	0.17	0.032		Pre-post	(1,57)	0.63	0.63	2.03	0.16	0.034
Balance group	(1,59)	2.60	2.60	1.37	0.25	0.023		Balance group	(1,57)	2.82	2.82	1.71	0.20	0.029
Type (EO, EC, VF)	**(2,58)**	**54.51**	**37.78**	**86.06**	**<0.001**	**0.593**		**Type (EO, EC, VF)**	**(2,56)**	**54.75**	**38.53**	**88.20**	**<0.001**	**0.607**
Pre-Post*Balance Group	**(1,59)**	**1.37**	**1.37**	**4.42**	**0.04**	**0.070**		**Diagnosis**	**(1,57)**	**18.44**	**18.44**	**11.19**	**0.001**	**0.164**
Pre-Post*Balance Group*Type	(2,58)	0.41	0.22	1.14	0.32	0.019		Pre-Post*Balance Group	(1,57)	1.20	1.20	3.89	0.05	0.064
								Pre-Post*Diagnosis	(1,57)	2.56 × 10^−5^	2.56 × 10^−5^	<0.001	0.99	<0.001
								Balance Group*Diagnosis	(1,57)	0.03	0.03	0.02	0.09	<0.001
								Pre-Post*Balance Group*Diagnosis	(1,57)	0.75	0.75	2.45	0.12	0.041
								Pre-Post*Balance Group*Diagnosis*Type	(2,56)	0.05	0.03	0.14	0.86	0.004

Significant results are bolded.

EO, eyes open; EC, eyes closed; VF, visual feedback.

#### Primary outcome: FA of CST

We did not find any statistically significant evidence supporting our hypothesis that FA in the CST would change as a function of balance training (see Supplementary Fig. 2 and Supplementary Table 1). Follow-up analyses that segmented the CST further confirmed a lack of change in this tract (see Supplementary Table 2).

#### Secondary outcome: Autism symptom severity

**Figure 3:**
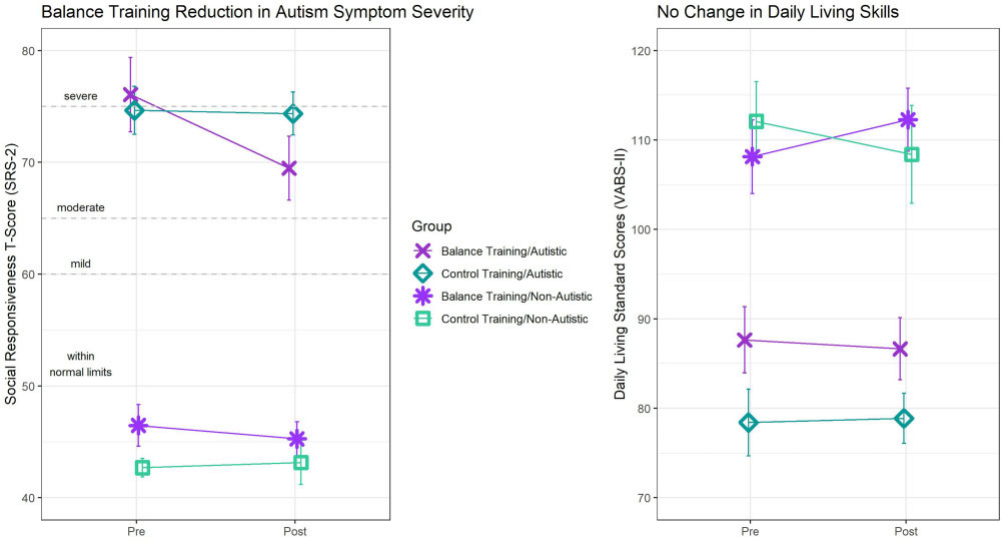
**Group-level associations with autism symptom severity and daily living skills.** Depiction of the group-level data (means ± one standard error) from the secondary outcome measures of (**A**) autism symptom severity measured by the total *t*-score of the Social Responsiveness Scale, 2nd edition (SRS-2), and (**B**) daily living skills, measured by daily living standard score of the Vineland Adaptive Behavior Scale, 2nd edition (VABS-2). The hypothesized balance-training-specific decreases in symptom severity were observed, but the hypothesized balance-training-specific increases in daily living skills were not observed.

Consistent with our hypotheses, Table 3 and Fig. 3 show a significant interaction between training group and pre-post measurement of autism symptom severity, *F*(1,60) = 6.06, *P* = 0.02, $\eta_{p}^{2}=0.092$, suggesting that the balance-training group demonstrated a steeper pre-post reduction of autism symptoms than the control group. The size of this effect was medium-to-large.^53^ There was one outlier in the autistic group, but even with this individual removed, results remained significant and indicated a medium sized effect.^53^ The three-way interaction from the follow-up 2 (balance/control) *×* 2 (pre/post) *×* 2 (autistic/non-autistic) mixed ANOVA was not significant, F(1,58) = 2.04, *P* = 0.16, $\eta_{p}^{2}=0.034$, suggesting that this effect was not different across diagnostic groups.

**Table 3:** ANOVA results for secondary outcomes (autism symptom severity and daily living skills), not considering diagnostic status (left) and considering diagnostic status (right)

**Autism symptom severity 2** × **2 ANOVA**								**Autism symptom severity 2** × **2** × **2 ANOVA**						
Predictor	*df*	Sum of squares	Mean square	*F*	*P*	$\eta_{p}^{2}$		Predictor	*df*	Sum of squares	Mean square	*F*	*P*	$\eta_{p}^{2}$
Pre-post	(1,60)	125.68	125.68	5.87	0.02	0.089		**Pre-post**	**(1,58)**	**111.16**	**111.16**	**5.52**	**0.02**	**0.087**
Balance group	(1,60)	13.51	13.51	0.024	0.88	<0.001		Balance group	(1,58)	11.18	11.18	0.08	0.78	0.001
Pre-Post*Balance Group	**(1,60)**	**129.88**	**129.88**	**6.06**	**0.02**	**0.092**		**Diagnosis**	**(1,58)**	**26177.94**	**26177.94**	**184.3**	**<0.001**	**0.761**
								Pre-Post * Balance Group	**(1,58)**	**121.14**	**121.14**	**6.02**	**0.02**	**0.094**
								Pre-Post*Diagnosis	(1,58)	72.25	72.25	3.59	0.06	0.058
								Balance Group*Diagnosis	(1,58)	167.6	167.6	1.18	0.28	0.020
								Pre-Post*Balance Group*Diagnosis	(1,58)	41.08	41.08	2.04	0.16	0.034
**VABS-II Daily Living Skill 2 × 2 ANOVA**								**VABS-II Daily Living Skill 2 × 2 × 2 ANOVA**						
**Predictor**	***df***	**Sum of squares**	**Mean square**	***F***	***P***	$\eta_{p}^{2}$		**Predictor**	***df***	**Sum of squares**	**Mean square**	***F***	***P***	$\eta_{p}^{2}$
Pre-post	(1,60)	0.04	0.04	0.001	0.98	<0.001		Pre-post	(1,58)	0.02	0.02	<0.001	0.99	<0.001
Balance group	(1,60)	978.03	978.03	1.28	0.26	0.021		Balance group	(1,58)	549.14	549.14	1.41	0.24	0.024
Pre-Post*Balance Group	(1,60)	58.11	58.11	0.8	0.38	0.013		**Diagnosis**	(1,58)	**22854.08**	**22854.08**	**58.65**	**<0.001**	**0.503**
								Pre-Post*Balance Group	(1,58)	77.3	77.3	1.07	0.31	0.018
								Pre-Post*Diagnosis	(1,58)	1.8	1.8	0.03	0.88	<0.001
								Balance Group*Diagnosis	(1,58)	557.15	557.15	1.43	0.24	0.024
								Pre-Post*Balance Group*Diagnosis	(1,58)	165.4	165.4	2.29	0.14	0.038
**W-ADL Daily Living Skill 2 × 2 ANOVA**								**W-ADL Daily Living Skills 2 × 2 × 2 ANOVA**						
**Predictor**	***df***	**Sum of squares**	**Mean square**	***F***	***P***	$\eta_{p}^{2}$		**Predictor**	***df***	**Sum of squares**	**Mean square**	***F***	***P***	$\eta_{p}^{2}$
Pre-post	(1,60)	1.97	1.97	0.34	0.56	0.006		Pre-post	(1,58)	1.70	1.70	0.29	0.59	0.005
Balance group	(1,60)	181.48	181.48	2.97	0.09	0.047		**Balance group**	**(1,58)**	**131.66**	**131.66**	**4.56**	**0.04**	**0.073**
Pre-Post*Balance Group	(1,60)	20.55	20.55	3.58	0.06	0.056		**Diagnosis**	**(1,58)**	**1989.36**	**1989.36**	**68.92**	**<0.001**	**0.543**
								Pre-Post*Balance Group	(1,58)	18.11	18.11	3.11	0.08	0.051
								Pre-Post*Diagnosis	(1,58)	2.79 × 10^−6^	2.79 × 10^−6^	<0.01	0.99	<0.001
								Balance Group*Diagnosis	(1,58)	12.25	12.25	0.42	0.52	0.007
								Pre-Post*Balance Group*Diagnosis	(1,58)	6.66	6.66	1.14	0.29	0.019

Significant results are bolded.

#### Secondary outcome: DLSs

Contrary to our hypothesis, Table 3 and Fig. 3 show that we did not find any statistically significant evidence that DLSs improved as a function of balance training.

#### Ancillary analyses

**Figure 4:**
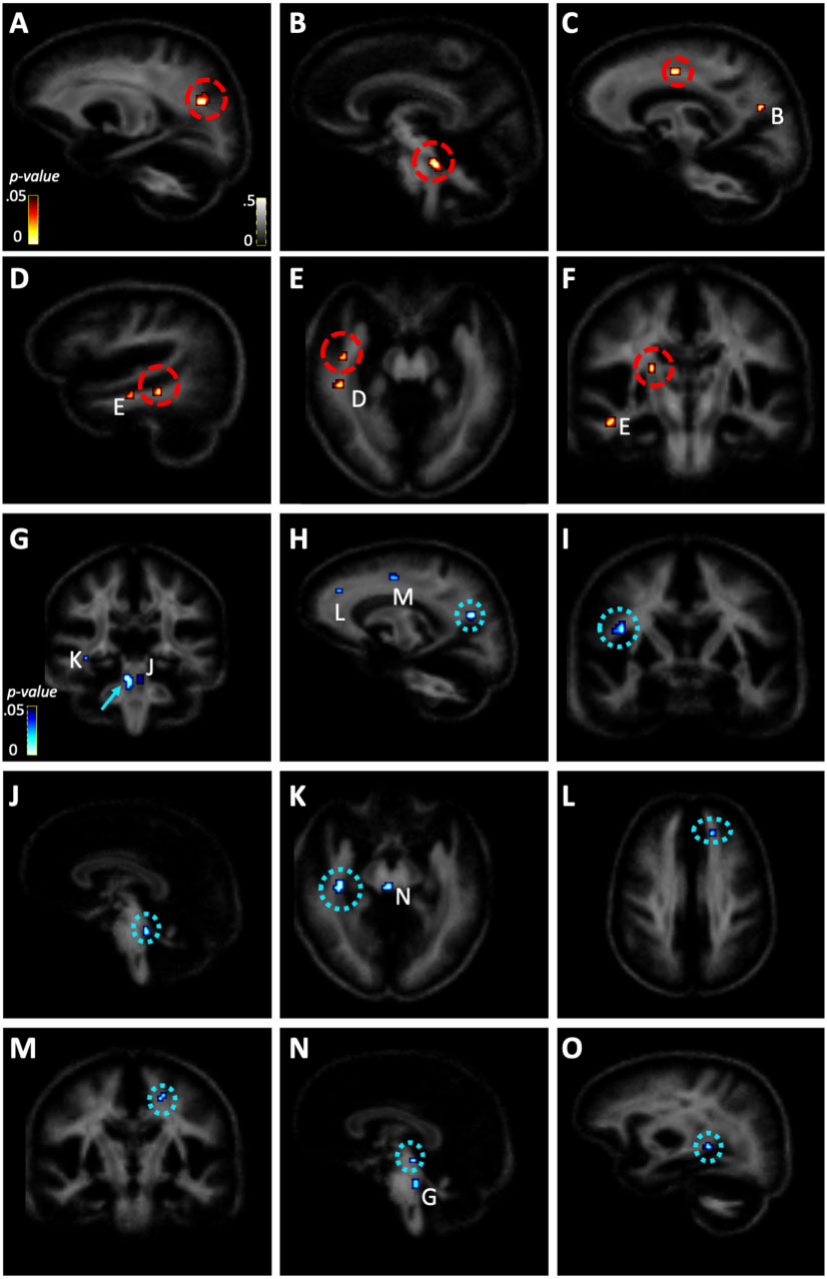
**Orientation dispersion index (ODI) voxel-based analysis results.** Two-way interactions between training group and pre-post measures depicted in red-yellow spectrum and three-way interactions among diagnostic group, training group, and pre-post measurement depicted in blue spectrum (*P* < 0.05, fdr-corrected and *k *≥* *5). Clusters with significant two-way interactions were found in the (**A**) left superior parietal/occipital white matter, (**B**) right SCP, (**C**) left cingulate gyrus, (**D and E**) right sagittal stratum, and (**F**) posterior limb of the internal capsule. Clusters with significant three-way interactions were found in the (**G**) right SCP, (**H**) superior parietal/occipital white matter, (**I**) primary motor cortex (posterior), (**J**) left SCP, (**K**) right sagittal stratum, (**L and M**) superior frontal white matter, (**N**) midline midbrain, and (**O**) right supramarginal gyrus white matter.

**Figure 5:**
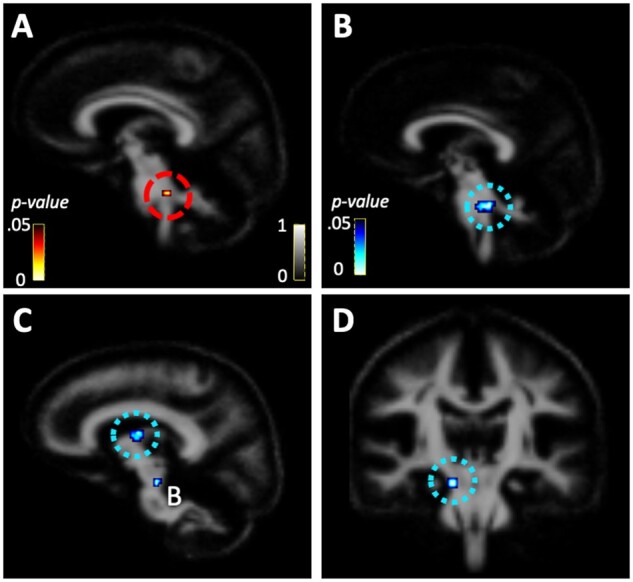
**Intracellular volume fraction (ICVF) voxel-based analysis results.** Two-way interactions between training group and pre-post measures depicted in red-yellow spectrum and three-way interactions among diagnostic group, training group, and pre-post measurement depicted in blue spectrum (*P* < 0.05, fdr-corrected and *k *≥* *5). One cluster with a significant two-way interaction was found in the (**A**) right SCP/medial lemniscus. Clusters with significant three-way interactions were found in the (**B**) right SCP/medial lemniscus, (**C**) right thalamic white matter, and (**D**) right cerebral peduncle.

Follow-up VBA investigated WM microstructure as a function of training group, time, and diagnostic group, after controlling for head motion, age and sex. We first examined two-way interactions between training group and time. Significant interactions between training group and time were found for ODI in the left superior parietal/occipital WM, left superior cerebellar peduncle (SCP), left cingulate gyrus WM, right sagittal stratum (two clusters in the temporal lobe) and right posterior limb of the internal capsule (PLIC) (fdr-corrected *P* < 0.05, *k *≥* *5 voxels) (Fig. 4 and Table 4). For ICVF, a significant interaction for training group and time was found in a cluster that centred on the right SCP and medial lemniscus (Fig. 5, Table 4). Graphical analysis of these interactions (Supplementary Figs. 3 and 4) demonstrate that these results were mostly reflective of increasing ODI in the balance-training group and decreasing ODI in the sedentary-control group. Graphical analysis of the two-way interaction between balance-training group and time in the SCP and medial lemniscus demonstrated decreasing ICVF in both the balance-training and sedentary-control groups.

**Table 4:** White matter (WM) regions reflecting change after balance training across both diagnostic groups (two-way interactions between balance-training group and pre-post measurements) as well as regions reflecting distinct diagnostic-group changes after balance training (three-way interactions among balance-training group, pre-post measurements, and diagnostic group)

Predictor	WM metric	Region	Right/ Left/Midline	Cluster size (voxels)	*X*	*Y*	*Z*	*P-*value
Pre-Post*Balance Group	Orientation dispersion index (ODI)	Superior parietal/occipital WM^a^	L	32	53	34	50	<0.001
		Superior cerebellar peduncle^a^	R	21	43	42	24	0.005
		Cingulate gyrus	L	8	53	64	55	0.007
		Sagittal stratum^a^	R	7	21	46	31	0.015
		Sagittal stratum	R	6	21	54	28	0.013
		Posterior limb of internal capsule	R	5	34	58	44	0.021
	Intracellular volume fraction (ICVF)	Superior cerebellar peduncle/medial lemniscus^a^	R	5	40	44	23	0.045
Pre-Post*Balance Group*Diagnosis	Orientation dispersion index (ODI)	Superior cerebellar peduncle	R	20	42	44	22	0.023
		Superior parietal/occipital WM	L	20	55	34	49	0.004
		Posterior primary motor cortex	R	8	24	66	43	0.018
		Superior cerebellar peduncle	L	8	46	43	24	0.010
		Sagittal stratum	R	8	25	50	31	0.012
		Superior frontal WM	L	5	54	65	57	0.026
		Superior frontal WM	L	5	52	84	45	0.042
		Midbrain	Midline	5	36	72	50	0.005
		Supramarginal WM	R	5	29	44	38	0.009
	Intracellular volume fraction (ICVF)	Medial lemniscus/superior cerebellar peduncle	R	28	42	46	21	0.014
		Thalamic WM	R	16	40	60	40	0.005
		Cerebral peduncle	R	5	38	50	27	0.030

a Overlapping regions where both a two-way and a three-way interaction was detected, thereby suggesting that diagnosis moderates the effects of this two-way interaction; *X*, *Y*, *Z* coordinates in MNI space; fdr*-*corrected *P* < 0.05, cluster threshold (*k*) ≥ 5 contiguous voxels.

When we examined the three-way interaction among diagnostic group, training group, and time, additional clusters emerged in ODI and ICVF (fdr-corrected *P* < 0.05, *k *≥* *5 voxels). We found that diagnostic status moderated all but two (right PLIC and left cingulate gyrus clusters) of the two-way interactions reported above. Additional ODI clusters for this three-way interaction emerged in the right posterior portion of the primary motor cortex, left SCP, left superior frontal WM (two clusters), midbrain and right supramarginal gyrus WM (Fig. 4, Table 4). Additional ICVF clusters for this three-way interaction emerged in the right thalamic WM and right cerebral peduncle (Fig. 5, Table 4). Graphical analysis of these interactions (Supplementary Figs. 3 and 4) demonstrated that the non-autistic group generally showed the expected increase of ODI and ICVF in the balance training condition, but the autistic group did not. The exception to this was ODI in the right cerebral peduncle and right sagittal stratum and ICVF in the right thalamic WM. The three-way, ODI interaction in the left superior parietal/occipital WM appeared to be driven primarily by different diagnostic effects in the sedentary-control group.

Because of the overlap in the VBA findings within the right SCP, a follow-up analysis examined how individual differences in one-foot balance times over the course of training (i.e. the slope of the training gains) accounted for post-training ICVF of the right SCP cluster, after controlling for pre-training ICVF of the right SCP cluster. Post-training ICVF of the right SCP cluster was significantly associated with the slope of the training gains, *b *=* *0.004, *se* = 0.002, *P* = 0.02. We found that the *adjusted R*^2^ increased by 9.9% when slope of training was included in the model, suggesting that an additional 9.9% of variance in post-training ICVF of this region was explained by individual differences in training progress.

Because autism symptom severity changes were found in the autistic balance-training group, further VBA explored pre-post changes in ODI and ICVF as a function of pre-post changes in autism symptom severity (SRS-2 raw scores, as age and sex were covariates in this model) within this group, while controlling for age, sex and head motion. All findings are shown in Supplementary Fig. 5 and Supplementary Table 4. For ODI, significant clusters were observed in the bilateral PLIC/caudate, right genu and left body of the corpus callosum, and left SCP, among others (*P* < 0.005, uncorrected, *k *≥* *5 voxels). For ICVF, significant clusters were observed in the right SCP, left thalamic WM, left splenium of the corpus callosum, left body of the corpus callosum/cingulate gyrus, among others (*P* < 0.005, uncorrected, *k *≥* *5 voxels). As can be seen in Supplementary Fig. 6, the locations of the right SCP cluster observed in the balance training interactions (both ODI and ICVF) and in the SRS-2 correlations are non-overlapping, although they were observed within the same tract.

## Discussion

The present study examined brain and behaviour changes as a function of a biofeedback-based balance training in autistic and non-autistic adolescents. The RCT design rigorously compared balance training and a sedentary video game control condition that were matched on key variables to control for non-intervention effects. The results suggest that the biofeedback-based balance training significantly improved balance and decreased parent-reported autism symptom severity. However, no training-related changes to DLSs were observed. Despite nearly identical behavioural improvements in balance, we found largely distinct patterns of structural brain changes in autistic participants compared to non-autistic participants in response to balance training. Taken together, these findings suggest that biofeedback-based balance training may target postural stability challenges, improve core autism symptoms and influence neurobiological change.

Balance improvements in participants were observed both during the in-game training and during outside-of-game postural sway measures. Both diagnostic groups significantly improved their one-footed and two-footed balance times over the course of the intervention training sessions. Even though the autistic group started the training with shorter balance times, autistic adolescents improved their balance at the same rate as the non-autistic adolescents, suggesting that this balance training renders similar outcomes in the same amount of time regardless of diagnostic status. Both autistic and non-autistic participants demonstrated substantial increases each session in one-footed poses, which equated to approximately 36 additional seconds of one-footed balance time by the end of training. One-footed gains may be particularly important because many daily living tasks require weight-shifting (i.e. walking on slippery surfaces, stepping into a shower, etc.) and because one-footed standing has been shown to be a pronounced challenge for autistic individuals.^15^^,^^54^ Outside of the game, participants in the balance-training group demonstrated a significant reduction in postural sway area during all visual-feedback conditions. Although postural sway during the pre-training assessment differed between the balance training and sedentary control groups, the reductions in the balance-training group were above-and-beyond that observed in the sedentary control group. Similar to previous findings with this training,^18^ these results suggest that in-game balance training appeared to transfer to outside-of-game balance improvements, demonstrating that balance gains made in a videogame-training context are likely to generalize to contexts outside of the video game.

Prior to this study, the links in the literature between motor challenges, autism symptom severity and DLSs^14–16^^,^^25^ raised the question of whether improvements in motor challenges may have subsequent reductions in autism symptom severity and/or improvements in DLSs. We did not observe the hypothesized pre-post changes in DLSs, suggesting that improving balance skills may not be sufficient to improve DLSs. This is in spite of the research that has found that balance is associated with DLSs, particularly in autistic individuals who have lower IQ scores.^55^ One possibility is that motor training may need to be paired with applied DLS training or occur for a longer period of time to have maximal benefit. However, we did observe significant decreases in autism symptom severity, such that autistic individuals assigned to the balance intervention moved from a severe symptom categorization to a moderate symptom categorization after training. In contrast, autistic participants assigned to the sedentary control group did not change in their symptom severity. We measured autism symptoms to gain insight into the nature of core autism symptoms and their relationship to motor challenges (not to try to eliminate autistic traits), and the present results importantly suggest that balance training may work to decrease parent-reported autism symptom severity. While future research will be needed to replicate this finding, a key advantage of the present study was the ability to examine potential neurobiological substrates that may underlie the relationship between improvements in balance and improvements in symptom severity. Follow-up, exploratory analyses further found that the decreases in autism symptom severity were associated with WM microstructure of the SCP, corpus callosum, PLIC and thalamus. These regions have been previously implicated in ASD^25^^,^^56–60^ and in balance training,^61^ independently. Given the small number of participants and lower statistical threshold in these follow-up exploratory analyses, future research with larger sample sizes of autistic participants will be needed to fully understand the brain basis of the autism symptom severity change as a function of intensive balance training. However, the association between microstructural neuroplasticity and improvements in symptom severity following balance training suggests that motor intervention may target neural structures that drive both motor ability and autism symptoms.

To our knowledge, this is the first study that reports structural neuroplasticity in response to motor training in autistic youth, and we found no evidence for our hypothesis that the microstructure of the CST, a descending motor tract, was impacted by balance training. However, follow-up, whole-brain analyses revealed neurite (WM axons and dendrite) plasticity in key brain areas that are candidate regions of interest for future research, including the SCP, primary motor cortex, thalamus, superior parietal/occipital WM and supramarginal gyrus. This lack of microstructural change in the CST coupled with the pattern of balance-training related neuroplasticity across the brain suggests that while balance training may not directly influence microstructure in primary motor pathways, it may affect microstructural change more globally, thus influencing motor ability. To our knowledge, this is the first finding of neurite-specific plasticity in response to balance training in humans, however balance studies in rodents have demonstrated dendritic structural plasticity in largely stable brain networks following balance (rotarod) paradigms.^62^^,^^63^ Additionally, the brain areas found to demonstrate balance-training related neurite plasticity have been frequently linked to balance and balance training using other structural imaging modalities, with the SCP being one of the most commonly implicated brain regions for balance.^61^ The SCP is the main WM tract by which information from the cerebellum transmits to the rest of the brain, connecting the cerebellum to the midbrain, basal ganglia^64^^,^^65^ and hypothalamus.^66^ WM microstructure of the SCP has been shown to be associated with proprioception in non-autistic adults,^67^ motor skills in autistic children,^68^ and motor skills, expressive language, and IQ in preterm-born children (some of whom were autistic).^69^ In the present study, we found corroborating evidence for the role of the SCP in motor and communication skills in that there were overlapping ODI and ICVF clusters in the right SCP associated with motor ability as well as a cluster in the superior portion of the SCP associated with autism symptom severity change. These clusters not only indicate the capacity for MR-detectible neurite plasticity in the brainstem following balance training but also may indicate that neurite orientation and axonal density in SCP particularly contribute to both motor ability and autism symptom severity. Moreover, follow-up analyses suggested that individual differences in balance training progress were highly predictive of individual differences in post-training ICVF of this region, after controlling for pre-training values. These findings converge to suggest that the SCP is involved in balance-related changes in autistic individuals, making this a particularly important tract to follow up on in future research.

Another key finding from the follow-up whole-brain analyses was the potentially unique neural responses to balance training in autistic individuals compared to non-autistic individuals, such that there were more diagnostic group distinctions than commonalities in neurite microstructural changes. Specifically, across the brain regions, there was a pattern for pre-post increases in ICVF and ODI in the balance-training group compared to the non-autistic sedentary-control group. However, this pattern was often not found in the autistic sedentary-control group, in spite of the fact that the training-related behavioural gains were similar in both groups. Interestingly, similar patterns of synaptic plasticity have been found in animals that underwent novel balance-specific training but not in animals that underwent general motor training,^62^ thus indicating that balance training may uniquely influence neurite structure. Furthermore, this influence of balance training on the brain may be different in autistic compared to non-autistic populations. As the brain has demonstrated neuroplasticity in response to novel motor experiences,^6^^,^^70^ it is quite possible that distinct the sensory and motor experiences of autistic individuals during the balance intervention may lead to unique brain structure–function relationships compared to non-autistic individuals. It is possible that balance training could target these unique structure–function relationships to induce a pattern of microstructural change across key WM areas that is specific to autistic individuals.

The present findings should be interpreted in light of limitations. While large enough to satisfy our power analysis estimates, the sample sizes of this study were modest, particularly given the heterogeneity observed in autistic individuals. Future studies should investigate this training in larger sample sizes. Also, of note, the present study used parent-reported measures to assess autism severity, following the majority of studies in the literature. However, a potentially even more powerful future test of the efficacy of this intervention will be to see if self-reported quality of life improves in autistic individuals, as quality of life is likely a more meaningful indicator of intervention success. Another limitation of the study is that the design did not include any post-training follow-up, making it unclear how long the training gains in balance and reductions in autism symptom severity were sustained. In terms of generalizability, this training was tested in autistic and non-autistic adolescents (13–17 years old) who communicated verbally and who generally had average IQ scores (although IQ scores varied). This means that these findings cannot be generalized to individuals who have co-occurring intellectual disability.

In conclusion, the present RCT suggests that biofeedback-based balance training significantly improved balance and decreased parent-reported autism symptom severity. However, no training-related changes to DLSs were observed, nor did we observe hypothesized changes in the microstructure of the CST. Instead, we found a wide range of balance-related structural changes across the brain, and these changes were often distinct in the autistic participants compared to the non-autistic participants. This finding suggests distinct microstructural changes in response to balance training in autistic individuals which may be indicative of distinct neural substrates of balance in ASD. Future research is encouraged to examine the SCP in response to balance training and symptom severity changes in autistic individuals, as the current study found overlapping findings in this brain region.

## Supplementary material

Supplementary material is available at *Brain Communications* online.

## Supplementary Material

fcab112_Supplementary_Data

## Abbreviations

ASD =: Autism Spectrum Disorder
BMI =: body mass index
COP =: centre of pressure
CST =: corticospinal tract
DLS =: daily living skill
DWI =: diffusion weighted imaging
FA =: fractional anisotropy
ICVF =: intracellular volume fraction
NODDI =: neurite orientation dispersion and density imaging
ODI =: orientation dispersion index
PLIC =: posterior limb of the internal capsule
RCT =: randomized controlled trial
SCP =: superior cerebellar peduncle
SRS-2 =: Social Responsiveness Scale, 2nd edition
T-SPOON =: tissue-specific, smoothing compensated
VABS-II =: Vineland Adaptive Behavior Scale, 2nd edition
VBA =: voxel-based analysis
W-ADL =: Waisman Activities of Daily Living Scale
WM =: white matter
