# Supplementary material for: Neurobiological and behavioural outcomes of biofeedback-based training in autism: a randomized controlled trial
Source: Brain Commun. 2021 May 27;3(3):fcab112. doi: 10.1093/braincomms/fcab112 (PMC8254423; doi:10.1093/braincomms/fcab112)
Supplement: fcab112_Supplementary_Data [file fcab112_supplementary_data.docx]

Supplementary Materials for **Neurobiological and Behavioral Outcomes of Balance Training in Autism: A Randomized Controlled Trial**

Olivia Surgent^1,2,^, Douglas C. Dean, the 3^rd1,3,4^, Andrew L. Alexander^1,4,5^, Olga I. Dadalko^1^, Jose Guerrero-Gonzalez ^1,4^, Desiree Taylor^1,6^, Emily Skaletski^1,6^, & Brittany G. Travers^1,6^

Author Affiliations: ^1^Waisman Center, University of Wisconsin-Madison, Madison, WI, USA ^, 2^Neuroscience Training Program, University of Wisconsin-Madison, Madison, WI, USA, ^3^Pediatrics, University of Wisconsin-Madison, Madison, WI, USA, ^4^Medical Physics, University of Wisconsin-Madison, Madison, WI, USA, ^5^Psychiatry, University of Wisconsin-Madison, Madison, WI, USA, ^6^Occupational Therapy Program in Kinesiology, University of Wisconsin-Madison, Madison, WI, USA

**Corresponding author’s contact information:**

Brittany G. Travers

Waisman Center

University of Wisconsin-Madison

1500 Highland Avenue, Room 435

Madison, WI 53705

Phone: (608) 263-0282

Fax: (608) 263-0529

Email: [btravers@wisc.edu](mailto:btravers@wisc.edu)

**Supplementary Methods**

**Sample Size**

G*power 3.1^1^ was used to estimate the a priori power of the present study. To detect a medium-sized (f=.25) interaction between two groups (training and control) and two measurements (pre- and post-training) with 80% power and an alpha of .05, we needed a total sample size of 34 participants (~17 in each group). For analyses that distinguished between the autistic and non-autistic groups using the same parameters as above, G*power is not able to detect power for a three-way interaction. However, as an approximation, we performed a power analysis for four groups (balance training in autistic individuals, balance training in non-autistic individuals, control condition in autistic individuals, control condition in non-autistic individuals) and two measurements (pre- and post-training), and we found that we would need a total sample size of 48 (~12 in each group).

**Study setting**

All study activities were performed at the University of Wisconsin’s Waisman Center in Madison, WI. The Waisman Center houses both research programs and a clinic dedicated to intellectual and developmental disabilities. All study activities were performed as part of the research project of the Waisman Center’s Motor and Brain Development Lab, which was separate from clinical services provided at the Waisman Center.

**Additional biofeedback-based video game training details**

When participating in the biofeedback-based video game training, the participant stood on the balance board in front of a 51” television that was mounted on the wall. Via the Kinect camera, the participant would see themselves on a blank television screen with 16 dots projected on their image to represent joint coordinates. A shadow representing the pose was also present on the screen. A research assistant adjusted this shadow to match the size of the participant. When the participant’s body was positioned within the shadow, all joint dots turned yellow. However, any joint dot not within the shadow turned red to alert the participant of the error. To reward the participant for holding the pose as long as possible, a background scene (mountain, beach, or tree cartoon landscapes) slowly appeared and became more saturated with each second the pose was maintained. When any two dots were not within the shadow, the background paused but would resume once all dots were back in the shadow. To individualize the training difficulty, the participant worked with the research assistant to set a goal for how long to hold each pose. This goal could range from 5-120 seconds, although the software allowed the participant to hold the pose for double the goal (i.e., a goal of 120 seconds could be held for up to 240 seconds).

**Similarity of interventions**

In both conditions, all participants completed three individualized trainings each week (60 minutes each) over the course of six weeks in the same lab space at the Waisman Center. Two research assistants facilitated each training session with the participant. Research assistants were trained on and facilitated both the balance-training and sedentary-control game protocols, thereby controlling for potential administrator effects between conditions. The sedentary-control games were matched as closely as possible to the balance-training games, using slow graphics and game pace for both conditions, a mid-session snack break, and Wii games (movement-based Wii games for the balance-training condition and sedentary Wii games for the sedentary-control condition). Participants in both conditions received $10 per hour for participation and $50 for each MRI scan.

**Pre-post postural stability measures**

Participants were instructed to stand on a Wii balance board (connected via Bluetooth to a Linux-based computer) with feet together on the center line of the board and arms crossed at the chest for 60 seconds. In the eyes-open condition, participants looked forward at a red dot on the wall. In the eyes-closed condition, participants closed their eyes while standing. In the visual-feedback condition, the computer screen was placed in front of the participant to view their center of pressure. Force on each of the four balance board sensors was recorded at 35 Hz and used to calculate center of pressure (COP), accounting for the distance between the four sensors on the Wii balance board.^2^ To control for possible movement at the beginning and end of the trial, we omitted the first and last five seconds. We *a priori* decided to use COP area.

**Diffusion weighted imaging preprocessing details**

DWI were denoised^3^ and corrected for Gibb’s ringing^4^ using MRtrix3.^5^ A brain mask was then created using *dwi2mask* from MRTrix3.^6^ Motion and eddy current corrections were performed using the *eddy* tool^7^ in FSL (version 6.0),^8^ with outlier replacement enabled.^9^ For a single DWI volume, if 10% or more of the slices were determined by *eddy* to be an outlier, the DWI volume was excluded from further analysis. We *a priori* decided that if 20% of the volumes were removed from a participant’s scan, we would exclude the participant from the analyses, but no participants met this threshold. Next, EPI distortion corrections were made using the FSL’s *fugue* tool.^10^ Bias correction was then performed using *dwibiascorrect* from MRTrix3^5^. Finally, an updated whole-brain mask was generated from the corrected DWI using the Brain Extraction Tool (BET) from FSL.^11^ This updated whole-brain mask was used for diffusion tensor fitting.

**Corticospinal tract (CST) delineation**

In order to delineate the bilateral CST, first fiber orientation distribution (FOD) function maps were created using *dwi2fod* from MRTrix3 with a multi-shell, multi-tissue constrained spherical deconvolution (MSMT-CSD) FOD estimation algorithm.^12^ FOD function maps were then registered to a study-specific population template. FOD function peaks were created using the *sh2peaks* tool from MRTrix3, with the normalized FOD function maps as input and a maximum of three peaks per voxel.^5,13^ *Tractse*g was then used to segment bundle start regions, segment bundle end regions, and create tract orientation maps (TOMs).^14^ The *tracking* tool was used to create CST specific tractograms from normalized FOD function peaks.^14,15^ The TOM trackings, bundle end regions, and normalized FA maps were then used to calculate mean FA across the entire CST. Follow-up analyses then examined the FA of the CST in 20 equally distanced segments along the streamlines^16^; <https://github.com/MIC-DKFZ/TractSeg/>).

**Statistical analysis**

**Balance time outcomes during game play.** Specifically, the linear mixed-effects model examined balance time (seconds) as function of session number (1-18), while accounting for random effects due to repeated measures. The linear fit was used to estimate each individual’s balance-training starting point (intercept) and the rate-of-change over the course of the training (slope).

**Imaging outcome analysis.** Because distal ends of the CST are more likely to have greater inter-subject variability due to registration artifacts,^17^ the superior and inferior most segments of the CST were excluded from analyses. Fdr-correction was used to control for multiple comparisons. The final model examined the dependent variable of CST FA as a result of the independent variables of time (pre/post), balance-training group, and their interaction, while accounting for tract laterality(left/right), tract segment (2-19), head motion, age at scan, sex, and random effects for intercepts due to repeated measures. All assumptions of this model were tested and found to be met. The effect of interest was the interaction between Time and Training Group. To examine whether the diagnostic groups differed in CST microstructure as a function of the intervention, we ran the same model as above with the addition of a diagnostic interaction effects with Time and Training Group as well as their three-way interaction. For this follow-up analysis, the effect of interest was the three-way interaction among Time, Training Group, and Diagnostic Group.

**TSPOON Processing.** The binary white matter mask was used to extract white matter-only maps of ODI and ICVF for each participant. These segmented maps and the white matter masks were spatially normalized to a study-specific population template space and smoothed with a 6mm FWHM Gaussian kernel. T-SPOON maps were then generated by dividing the normalized, smoothed, segmented ODI and ICVF maps by the normalized smoothed white matter mask for each participant. A population-based white matter mask was created by taking the average of the normalized smoothed white matter masks. To further prevent partial volume effects, VBA analyses were restricted to voxels within this average white matter mask that had a value above 0.50, indicating a greater than 50% chance of white matter in each voxel across participants.

**Structural Localization.** In order to identify brain regions where significant clusters were located, we warped the JHU atlas ^18^ to our study-specific population template. Atlas labels were used to localize clusters within white matter tracts in the brain. If a cluster spanned multiple JHU-defined regions, the cluster was considered to be a part of the JHU-defined region that contained the majority of voxels within the cluster.

**Supplementary Results**

**Primary Outcome: FA of the CST**

Contrary to our hypothesis, there was not a statistically significant relationship between balance training and CST FA. The three-way interaction from the follow-up linear mixed effect analysis accounting for diagnostic group was also not significant, *b* = -0.01, *se* = 0.01, *p* = .11, suggesting that this null effect was consistent across diagnostic groups. Because the CST is a long, bilateral tract, follow-up analyses divided each CST into 20 segments and examined the predicted two-way and three-way interactions within each segment (omitting the most distal segments). As can be seen in Supplementary Table 2, after controlling for multiple comparisons, there were no significant interactions of interest in any of the segments.

**Secondary outcome: Daily living skills**

Contrary to our hypotheses, there were no statistically significant effects of balance training on W-ADL daily living skills. However, there was a small-to-medium-sized^53^ but not significant interaction between training group and pre-post measurement on W-ADL daily living skills, *F*(1,60) = 3.58, *p* = .06, *𝝶_p_^2^* = .056. There was no evidence of an interaction between training group and pre-post measurement on the VABS-II daily living skills standard scores, *F*(1,60) = 0.80, *p* = .38, *𝝶_p_^2^* = .013 (Figure 4B), suggesting that the balance-training group demonstrated a similar pre-post changes in daily living skills compared to the control group. The three-way interaction from the follow-up mixed ANOVA including diagnostic group was also not significant for the W-ADL, *F*(1,58) = 1.14, *p* = .29, *𝝶_p_^2^* = .019, nor for the VABS-II scores, *F*(1,58) = 2.29, *p* = .14, *𝝶_p_^2^* = .038, suggesting that this lack of effect was consistent across diagnostic groups.

**Ancillary analyses: SCP clusters**

We found the SCP to be a critical region of interest for future studies as clusters in the SCP were associated with both balance skills and reductions in symptom severity. At least 50% of the voxels in each right SCP cluster were within the JHU-defined right SCP region. Specifically, the ICVF and ODI clusters associated with balance had 60% (3/5), 80% (17/21) of voxels within the JHU-defined right SCP, respectively. 53% (8/15) of the voxels within the cluster associated with SRS change were in the superior right SCP.

**Supplementary Figure 1**. Participant flow CONSORT diagram. Note, eligibility assessment numbers are not presented because the eligibility screenings were not recorded or retained.


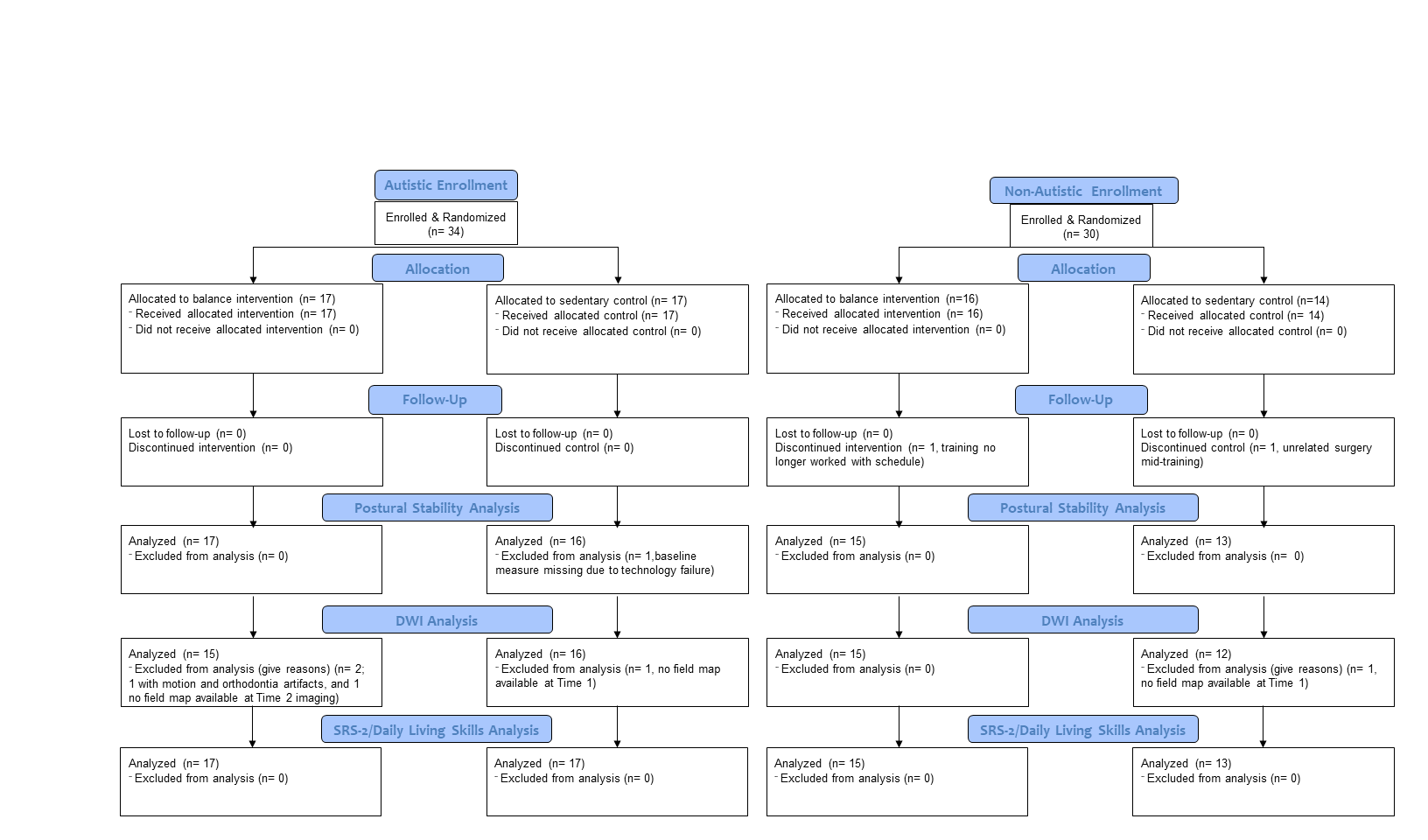


**Supplementary Figure 2.** Tractography of the bilateral corticospinal tracts (CST) in a representative participant and depiction of the group-level (means±one standard error) fractional anisotropy measure averaged across these bilateral tracts for each individual. There was a small-sized but non-significant three-way interaction among diagnostic group, training group, and pre-post measurement. However, an examination of the means shows that this appeared to be driven by autistic participants in the sedentary-control condition, rather than by balance training.

**
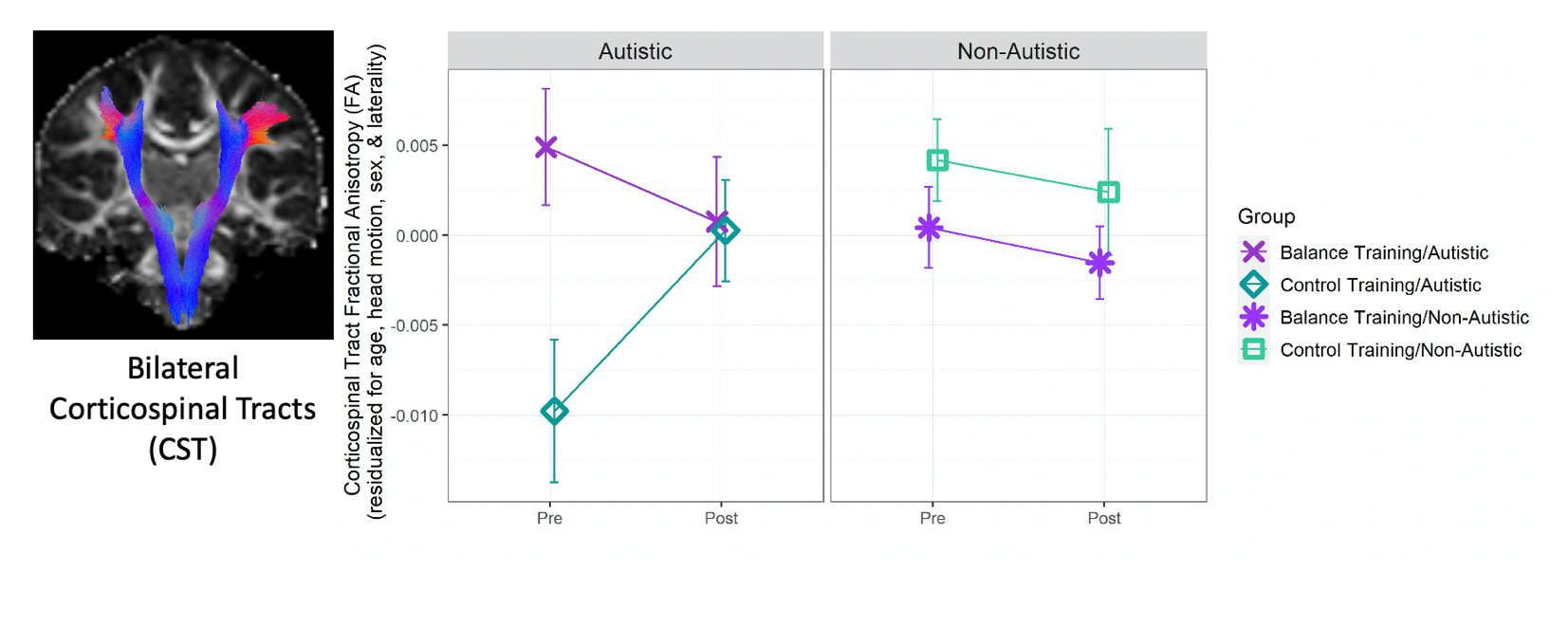
**

**Supplementary Figure 3**. Graphical analysis of interactions for orientation dispersion index (ODI) (corresponding to clusters in Figure 4). Group means±standard error are shown.


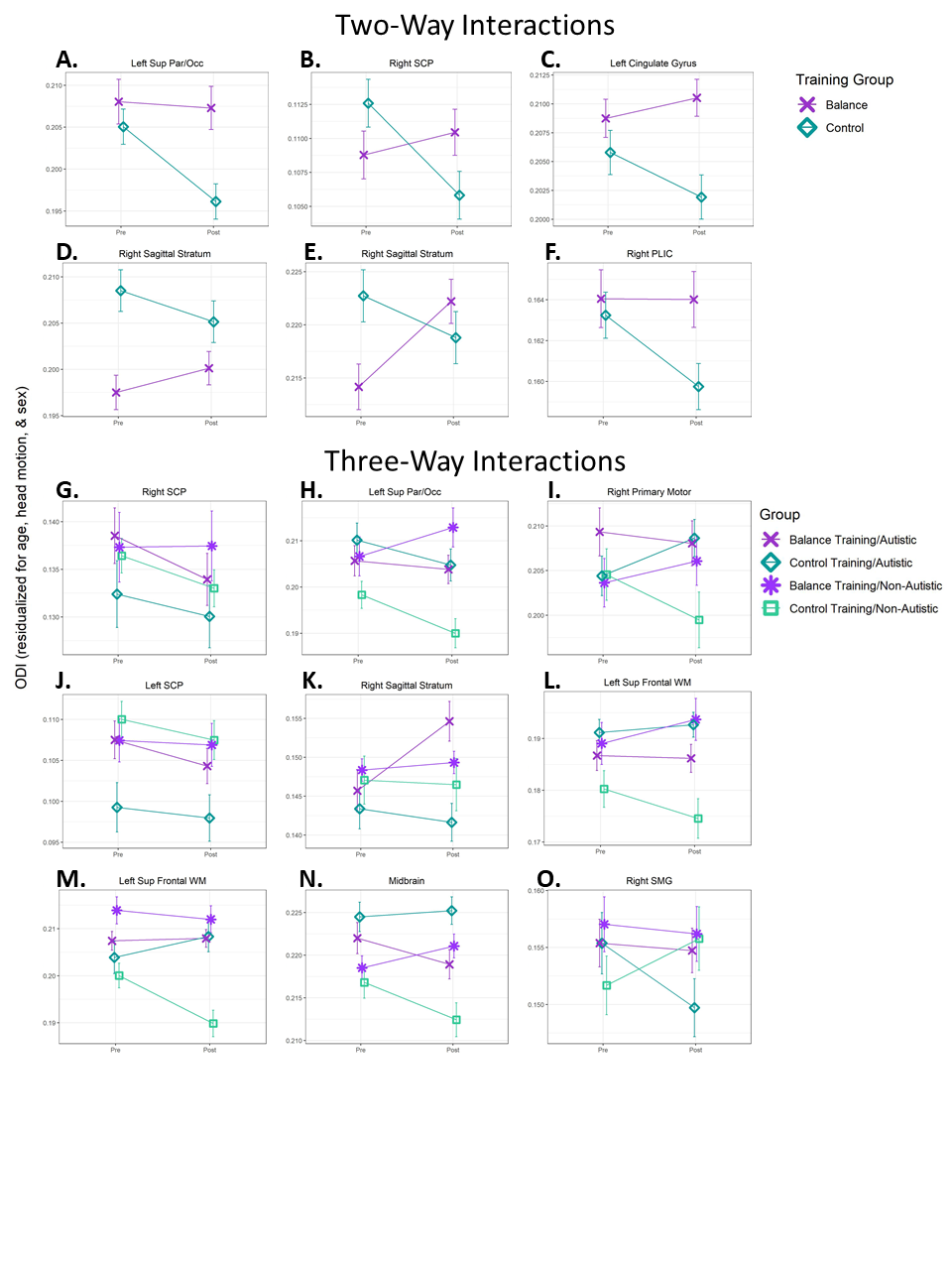


**Supplementary Figure 4**. Graphical analysis of interactions for intracellular volume fraction (ICVF) (corresponding to clusters in Figure 5). Group means±standard error are shown.


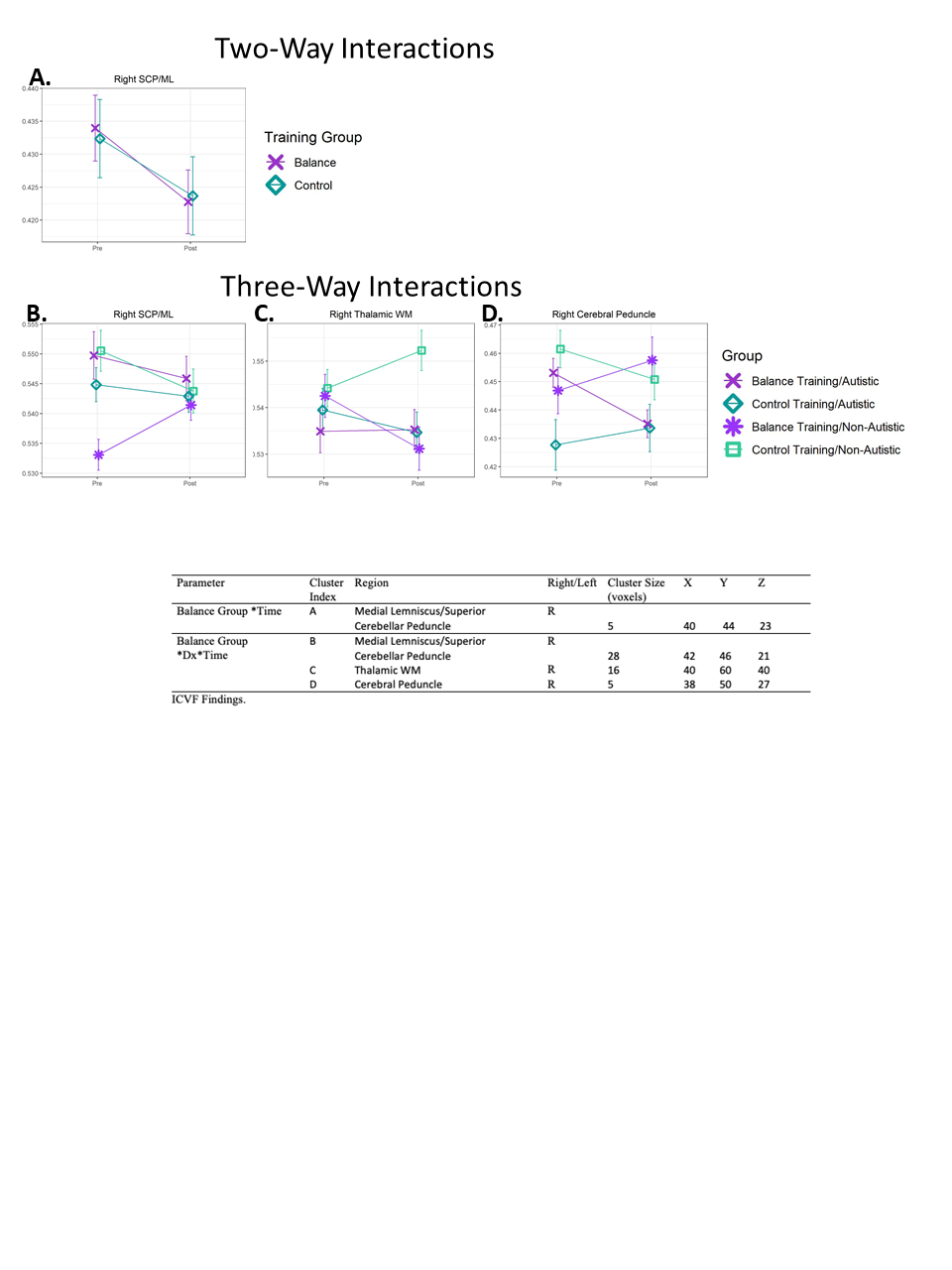


**Supplementary Figure 5.** Voxel-based analysis results examining pre-post changes in white matter (WM) as a function of pre-post changes in autism symptom severity (SRS-2 raw scores) in intracellular volume fraction (ICVF; blue spectrum) and orientation dispersion index (ODI; red-yellow spectrum). Analysis only occurred in the balance-training group of autistic participants, *p*<.005, uncorrected and *k*≥5.


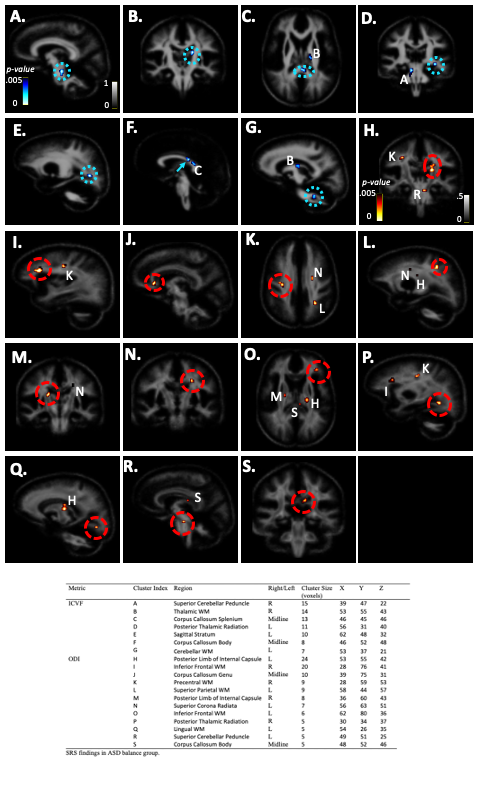


**Supplementary Figure 6.** Locations of the right superior cerebellar peduncle (SCP) clusters found across this study. The blue cluster shows where changes in the right SCP intracellular volume fraction (ICVF) were related to changes in autism symptom severity in the autistic, balance-training group (p<.005, uncorrected and k≥5). The red cluster shows where there was a two-way interaction between training group and pre-post measures and a three- way interaction among diagnostic group, training group, and pre-post measurement in ICVF of the right SCP (p<.05, fdr-corrected and k≥5). The green cluster shows where there was a two-way interaction between training group and pre-post measures and a three-way interaction among diagnostic group, training group, and pre-post measurement in orientation dispersion index (ODI) of the right SCP (p<.05, fdr-corrected and k≥5). While there is spatial overlap between the green and red clusters, there is not overlap with the blue cluster.


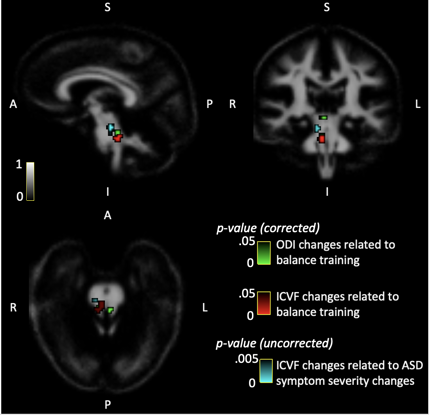


**Supplementary Table 1**. Bilateral corticospinal tract (CST) fractional anisotropy (FA) as a function of pre-post assessment and balance group (2-way interaction model) and as a function of pre-post assessment, balance group, and diagnosis (3-way interaction model). All models co-varied for tract side (left/right), head motion, age, and sex. Significant effects are bolded.

| **CST FA: 2-way interaction model** | | | | | |  | **CST FA: 3-way interaction model** | | | | |
| --- | --- | --- | --- | --- | --- | --- | --- | --- | --- | --- | --- |
| **Predictor** | ***b*** | **se** | **t-value** | ***p*** |  | | **Predictor** | ***b*** | **se** | **t-value** | ***p*** |
| Pre-Post | .004 | .003 | 1.47 | .14 |  | | Pre-Post | -.002 | .004 | -.48 | .63 |
| Balance Group | .006 | .005 | 1.17 | .25 |  | | Balance Group | -.004 | .007 | -.51 | .62 |
| **Side (Left/Right)** | **-.011** | **.002** | **-6.15** | **<.001** |  | | **Side (Left/Right)** | **-.011** | **.002** | **-6.17** | **<.001** |
| Head motion | -.020 | .014 | -1.46 | .15 |  | | Head motion | -.015 | .014 | -1.06 | .29 |
| Age | .002 | .002 | 1.20 | .23 |  | | Age | .002 | .002 | 1.22 | .23 |
| Sex | .009 | .006 | 1.43 | .16 |  | | Sex | .008 | .007 | 1.19 | .24 |
| Pre-Post * Balance Group | -.007 | .004 | -1.83 | .07 |  | | Pre-Post * Balance Group | -.0002 | .005 | -.03 | .97 |
|  |  |  |  |  |  | | **Pre-Post*Diagnosis** | **.010** | **.005** | **1.96** | **.05** |
|  |  |  |  |  |  | | Balance Group * Diagnosis | .017 | .010 | 1.75 | .09 |
|  |  |  |  |  |  | | Pre-Post * Balance Group * Diagnosis | -.012 | .007 | -1.62 | .11 |

**Supplementary Table 2**. Segmented bilateral corticospinal tract (CST) fractional anisotropy (FA) as a function of pre-post assessment and balance group (2-way interaction model) and as a function of pre-post assessment, balance group, and diagnosis (3-way interaction model). All models co-varied for tract side (left/right), head motion, age, and sex and used FDR to control for multiple comparisons.

|  | **CST FA: 2-way interaction model**  (**Training Group x Time)** | | | | **CST FA: 2-way interaction model (Diagnosis x Training Group x Time)** | | | |
| --- | --- | --- | --- | --- | --- | --- | --- | --- |
| **CST Segment**  **(inferior to superior)** | **b** | **se** | **t-value** | **p-value**  **(FDR-adjusted)** | **b** | **se** | **t-value** | **p-value**  **(FDR-adjusted)** |
| 2 | -0.014 | 0.014 | -0.96 | 0.87 | -0.034 | 0.029 | -1.18 | 0.39 |
| 3 | -0.014 | 0.012 | -1.17 | 0.86 | -0.037 | 0.025 | -1.50 | 0.35 |
| 4 | -0.002 | 0.012 | -0.15 | 0.88 | 0.012 | 0.025 | 0.48 | 0.67 |
| 5 | 0.002 | 0.007 | 0.24 | 0.88 | 0.009 | 0.014 | 0.63 | 0.59 |
| 6 | -0.003 | 0.007 | -0.47 | 0.88 | -0.005 | 0.014 | -0.35 | 0.73 |
| 7 | 0.002 | 0.008 | 0.29 | 0.88 | -0.017 | 0.017 | -1.02 | 0.43 |
| 8 | -0.002 | 0.008 | -0.21 | 0.88 | -0.025 | 0.016 | -1.57 | 0.35 |
| 9 | -0.005 | 0.009 | -0.57 | 0.88 | -0.030 | 0.018 | -1.69 | 0.35 |
| 10 | -0.006 | 0.010 | -0.55 | 0.88 | -0.041 | 0.020 | -2.03 | 0.35 |
| 11 | -0.003 | 0.008 | -0.38 | 0.88 | -0.034 | 0.016 | -2.11 | 0.35 |
| 12 | -0.002 | 0.008 | -0.23 | 0.88 | -0.028 | 0.017 | -1.66 | 0.35 |
| 13 | -0.008 | 0.008 | -1.07 | 0.86 | -0.026 | 0.016 | -1.66 | 0.35 |
| 14 | -0.008 | 0.009 | -0.86 | 0.88 | -0.016 | 0.018 | -0.90 | 0.48 |
| 15 | -0.009 | 0.007 | -1.24 | 0.86 | -0.016 | 0.014 | -1.12 | 0.40 |
| 16 | -0.008 | 0.007 | -1.18 | 0.86 | -0.016 | 0.013 | -1.19 | 0.39 |
| 17 | -0.007 | 0.006 | -1.09 | 0.86 | 0.010 | 0.013 | 0.78 | 0.52 |
| 18 | 0.004 | 0.008 | 0.49 | 0.88 | 0.020 | 0.015 | 1.31 | 0.39 |
| 19 | -0.016 | 0.012 | -1.35 | 0.86 | 0.031 | 0.024 | 1.28 | 0.39 |

**Supplementary Table 3.** White matter (WM) regions reflecting pre-post balance training changes in autism symptom severity (SRS-2 raw scores) within autistic participants.

| **Metric** | **Cluster Index** | **Region** | **Right/Left/Midline** | **Cluster Size (voxels)** | **X** | **Y** | | **Z** | | |
| --- | --- | --- | --- | --- | --- | --- | --- | --- | --- | --- |
| ICVF | A | Superior Cerebellar Peduncle | R | 15 | 39 | | 47 | | 22 |  |
|  | B | Thalamic WM | R | 14 | 53 | | 55 | | 43 |  |
|  | C | Corpus Callosum Splenium | Midline | 13 | 46 | | 45 | | 46 |  |
|  | D | Posterior Thalamic Radiation | L | 11 | 56 | | 31 | | 40 |  |
|  | E | Sagittal Stratum | L | 10 | 62 | | 48 | | 32 |  |
|  | F | Corpus Callosum Body | Midline | 8 | 46 | | 52 | | 48 |  |
|  | G | Cerebellar WM | L | 7 | 53 | | 37 | | 21 |  |
| ODI | H | Posterior Limb of Internal Capsule | L | 24 | 53 | | 55 | | 42 |  |
|  | I | Inferior Frontal WM | R | 20 | 28 | | 76 | | 41 |  |
|  | J | Corpus Callosum Genu | Midline | 10 | 39 | | 75 | | 31 |  |
|  | K | Precentral WM | R | 9 | 28 | | 59 | | 53 |  |
|  | L | superior parietal WM | L | 9 | 58 | | 44 | | 57 |  |
|  | M | Posterior Limb of Internal Capsule | R | 8 | 36 | | 60 | | 43 |  |
|  | N | Superior Corona Radiata | L | 7 | 56 | | 63 | | 51 |  |
|  | O | Inferior Frontal WM | L | 6 | 62 | | 80 | | 36 |  |
|  | P | Posterior Thalamic Radiation | R | 5 | 30 | | 34 | | 37 |  |
|  | Q | Lingual WM | L | 5 | 54 | | 26 | | 35 |  |
|  | R | Superior Cerebellar Peduncle | L | 5 | 49 | | 51 | | 25 |  |
|  | S | Corpus Callosum Body | Midline | 5 | 48 | | 52 | | 46 |  |

Note: X,Y,Z coordinates in MNI space; fdr*-*corrected *p* < .05, cluster threshold (k) ≥ 5 contiguous voxels.

**References**

1. Faul F, Erdfelder E, Buchner A, Lang A-G. Statistical power analyses using G*Power 3.1: Tests for correlation and regression analyses. *Behavior Research Methods*. 2009;41(4):1149-1160. doi:10.3758/BRM.41.4.1149

2. Bartlett HL, Ting LH, Bingham JT. Accuracy of force and center of pressure measures of the Wii Balance Board. *Gait & Posture*. 2014;39(1):224-228. doi:10.1016/j.gaitpost.2013.07.010

3. Veraart J, Fieremans E, Jelescu IO, Knoll F, Novikov DS. Gibbs ringing in diffusion MRI: Gibbs Ringing in Diffusion MRI. *Magn Reson Med*. 2016;76(1):301-314. doi:10.1002/mrm.25866

4. Kellner E, Dhital B, Kiselev VG, Reisert M. Gibbs-ringing artifact removal based on local subvoxel-shifts: Gibbs-Ringing Artifact Removal. *Magn Reson Med*. 2016;76(5):1574-1581. doi:10.1002/mrm.26054

5. Tournier J-D, Smith R, Raffelt D, et al. MRtrix3: A fast, flexible and open software framework for medical image processing and visualisation. *NeuroImage*. 2019;202:116137. doi:10.1016/j.neuroimage.2019.116137

6. Dhollander T, Raffelt D, Connelly A. Unsupervised 3-tissue response function estimation from single-shell or multi-shell diffusion MR data without a co-registered T1 image. In: Vol pp. 5. ; 2016.

7. Andersson JLR, Sotiropoulos SN. An integrated approach to correction for off-resonance effects and subject movement in diffusion MR imaging. *NeuroImage*. 2016;125:1063-1078. doi:10.1016/j.neuroimage.2015.10.019

8. Jenkinson M, Beckmann CF, Behrens TEJ, Woolrich MW, Smith SM. FSL. *NeuroImage*. 2012;62(2):782-790. doi:10.1016/j.neuroimage.2011.09.015

9. Andersson JLR, Graham MS, Zsoldos E, Sotiropoulos SN. Incorporating outlier detection and replacement into a non-parametric framework for movement and distortion correction of diffusion MR images. *NeuroImage*. 2016;141:556-572. doi:10.1016/j.neuroimage.2016.06.058

10. Smith SM, Jenkinson M, Woolrich MW, et al. Advances in functional and structural MR image analysis and implementation as FSL. *NeuroImage*. 2004;23:S208-S219. doi:10.1016/j.neuroimage.2004.07.051

11. Smith SM. Fast robust automated brain extraction. *Hum Brain Mapp*. 2002;17(3):143-155. doi:10.1002/hbm.10062

12. Jeurissen B, Tournier J-D, Dhollander T, Connelly A, Sijbers J. Multi-tissue constrained spherical deconvolution for improved analysis of multi-shell diffusion MRI data. *NeuroImage*. 2014;103:411-426. doi:10.1016/j.neuroimage.2014.07.061

13. Jeurissen B, Leemans A, Tournier J-D, Jones DK, Sijbers J. Investigating the prevalence of complex fiber configurations in white matter tissue with diffusion magnetic resonance imaging: Prevalence of Multifiber Voxels in WM. *Hum Brain Mapp*. 2013;34(11):2747-2766. doi:10.1002/hbm.22099

14. Wasserthal J, Neher PF, Hirjak D, Maier-Hein KH. Combined tract segmentation and orientation mapping for bundle-specific tractography. *Medical Image Analysis*. 2019;58:101559. doi:10.1016/j.media.2019.101559

15. Wasserthal J, Neher P, Maier-Hein KH. TractSeg - Fast and accurate white matter tract segmentation. *NeuroImage*. 2018;183:239-253. doi:10.1016/j.neuroimage.2018.07.070

16. Chandio BQ, Risacher SL, Pestilli F, et al. Bundle analytics, a computational framework for investigating the shapes and profiles of brain pathways across populations. *Sci Rep*. 2020;10(1):17149. doi:10.1038/s41598-020-74054-4

17. Yeatman JD, Dougherty RF, Myall NJ, Wandell BA, Feldman HM. Tract Profiles of White Matter Properties: Automating Fiber-Tract Quantification. Beaulieu C, ed. *PLoS ONE*. 2012;7(11):e49790. doi:10.1371/journal.pone.0049790

18. Oishi K, Faria A, Jiang H, et al. Atlas-based whole brain white matter analysis using large deformation diffeomorphic metric mapping: Application to normal elderly and Alzheimer’s disease participants. *NeuroImage*. 2009;46(2):486-499. doi:10.1016/j.neuroimage.2009.01.002
